# Structural basis of the methylation specificity of R.DpnI

**DOI:** 10.1093/nar/gku546

**Published:** 2014-06-25

**Authors:** Karolina Mierzejewska, Wojciech Siwek, Honorata Czapinska, Magdalena Kaus-Drobek, Monika Radlinska, Krzysztof Skowronek, Janusz M. Bujnicki, Michal Dadlez, Matthias Bochtler

**Affiliations:** 1 International Institute of Molecular and Cell Biology, Trojdena 4, 02-109 Warsaw, Poland; 2 Institute of Biochemistry and Biophysics PAS, Pawinskiego 5a, 02-106 Warsaw, Poland; 3 Institute of Microbiology, Faculty of Biology, University of Warsaw, Miecznikowa 1, 02-096 Warsaw, Poland; 4 Institute of Molecular Biology and Biotechnology, Adam Mickiewicz University, Umultowska 89, 61-614 Poznan, Poland

## Abstract

R.DpnI consists of N-terminal catalytic and C-terminal winged helix domains that are separately specific for the Gm6ATC sequences in Dam-methylated DNA. Here we present a crystal structure of R.DpnI with oligoduplexes bound to the catalytic and winged helix domains and identify the catalytic domain residues that are involved in interactions with the substrate methyl groups. We show that these methyl groups in the Gm6ATC target sequence are positioned very close to each other. We further show that the presence of the two methyl groups requires a deviation from B-DNA conformation to avoid steric conflict. The methylation compatible DNA conformation is complementary with binding sites of both R.DpnI domains. This indirect readout of methylation adds to the specificity mediated by direct favorable interactions with the methyl groups and solvation/desolvation effects. We also present hydrogen/deuterium exchange data that support ‘crosstalk’ between the two domains in the identification of methylated DNA, which should further enhance R.DpnI methylation specificity.

## INTRODUCTION

Methylation is known to regulate DNA binding and cleavage in a wide variety of biological contexts, either positively or negatively. Discrimination against methylated DNA can be readily explained in terms of the high penalty for clashes of the methyl group with the DNA-binding site of the protein. Selectivity for methylated DNA is much harder to explain and may not follow a universal mechanism. For different model systems, CH···O hydrogen-bonding interactions (1), cation–π interactions (2) and solvation/desolvation effects (3,4) have all been cited to explain the preference of methylated DNA.

Restriction-modification systems provide excellent opportunities to study effects of methylation on DNA binding and cleavage. Their function is based on the fact that nuclease-mediated cleavage is regulated by methylation. Moreover, endonucleases tend to form tight complexes with their substrates, which are well amenable to biochemical and structural studies.

Most restriction systems protect bacteria against the invasion of foreign DNA by marking the ‘self’ DNA by methylation and degrading DNA that is identified as ‘non-self’ because of a lack of this modification (5). However, some endonucleases that protect bacteria against invading DNA rely on a reversed relationship between methylation/non-methylation and self/non-self. The ‘reversal’ of the role of DNA modification can plausibly be attributed to the ‘arms race’ between bacteria and phages. According to this theory, phages have responded to the selective pressure against non-methylated DNA by acquiring methylation (either in a previous host or through acquisition of a methyltransferase). In turn, bacteria have responded by the evolution of modification-specific restriction endonucleases and, when necessary, the loss of the cognate methyltransferases (6). The structural basis of the modification specificity of these endonucleases is not well understood.

Methylation-specific restriction endonucleases belonging to the large group of type II enzymes are classified as type IIM, where the ‘M’ stands for ‘modification’ or ‘methylation’ (7), and form a fairly diverse group. 5-methylcytosine (m5C) specific restriction endonucleases include the GTP-dependent McrBC (8), as well as the nucleoside-triphosphate-independent R.MspJI (9) and Mrr (10). Recently, much progress has been made to elucidate the structural basis of the specificity of these enzymes (11,12). In contrast, 6-methyladenine (m6A) dependent restriction endonucleases have received less attention, despite the widespread use of R.DpnI for site-directed mutagenesis (13).

The prototypical m6A-dependent endonuclease R.DpnI was originally isolated from *Streptococcus pneumoniae*. *In vivo* the protein protects the Dam−, R.DpnI+ bacteria against phages that have been propagated on Dam+ hosts. The biological activity is due to the specificity of R.DpnI for Dam-methylated (Gm6ATC) sites (14). R.DpnI cleaves its target sequence with high efficiency if both DNA strands are methylated and with lesser efficiency if only one strand is modified (15).

In previous work, we have biochemically and structurally characterized R.DpnI (16). We have shown that the enzyme is a type IIE restriction endonuclease and consists of an N-terminal catalytic domain of the PD-(D/E)XK type (residues 1-182) and a C-terminal winged helix domain (residues 183-254). Both domains are sensitive to DNA sequence and methylation status. In the previously solved crystal structure, there were two copies of R.DpnI-DNA complex in the asymmetric unit. In both cases, the DNA was bound to the winged helix domain of the enzyme, leaving its catalytic domain in a substrate-free form with a disordered active site region (16).

Here, we report new studies that shed light on the methylation specificity of R.DpnI. We present a crystal form of R.DpnI with one molecule of the enzyme and two target DNA duplexes in the asymmetric unit. One oligoduplex is bound to the winged helix domain as previously reported. The other duplex binds to the catalytic domain in a conformation that is posed for cleavage. We characterize the involvement of residues forming the methyl binding cleft of the catalytic domain by site-directed mutagenesis. We further show that the methylation specificity of the R.DpnI domains likely results from a combination of direct favorable interactions with the methyl groups, solvation/desolvation effects and indirect effects imposed by the proximity of the methyl groups on DNA conformation. Finally, we present hydrogen/deuterium exchange experiments indicating a ‘crosstalk’ between the winged helix and catalytic domains, which is likely to enhance the methylation specificity of R.DpnI.

## MATERIALS AND METHODS

### Protein and DNA preparation

The *dpnI* gene was expressed and the R.DpnI protein purified as previously described (16). In short detail, the gene in fusion with N-terminal His-tag was expressed in *Escherichia coli* ER2925 Dam− strain lysogenized with DE3 element. The protein was purified by nickel affinity chromatography. The His-tag was cleaved off with the PreScission protease and a second chromatography step on an ion exchange MonoS column (GE Healthcare) was performed. Finally, the enzyme was subjected to buffer exchange on a PD-10 column (GE Healthcare). Protein variants with amino acid substitutions were prepared using the standard QuikChange site-directed mutagenesis protocol (17) and purified analogously to the wild-type enzyme (wt R.DpnI). R.DpnI and its variants were analyzed for protein purity by Coomassie-stained SDS-PAGE (1 μg per lane).

Self-complementary oligonucleotide 5′-CTGGm6ATCCAG-3′ used in the crystallization trials was synthesized on ASM 800 DNA synthesizer BIOSSET according to the manufacturer's specifications. The oligonucleotides were then deprotected, purified on HPLC C18 column and lyophilized. The obtained samples were dissolved in nuclease-free milliQ water and annealed by slow cooling from 95°C to 4°C to form duplex DNA.

### Crystallization

R.DpnI was concentrated to 12.5 mg/ml (0.42 mM) in 15% (v/v) glycerol, 50 mM PIPES pH 7.0, 150 mM NaCl, 10 mM β-mercaptoethanol, 50 mM arginine, 50 mM glutamic acid and 5 mM CaCl_2_. The protein and DNA duplex were mixed in 1:3 molar ratio to form a tight complex. Crystals were grown at 4°C by the sitting drop vapor diffusion method using equal amounts of protein-DNA mix and crystallization buffer (160 mM calcium acetate, 80 mM sodium cacodylate pH 6.5, 14.4% w/v PEG 8000, 20% v/v glycerol, 10 mM spermidine). Prior to data collection, crystals were flash-cryocooled to 100 K. The diffraction data (2.35 Å) was collected at 1.23953 Å wavelength at the MX2 beamline of the PETRA storage ring (DESY, Hamburg). The data collection statistics are listed in Supplementary Table S1.

### Structure determination and refinement

The program DIBER (18) predicted with high confidence that the crystals obtained from the R.DpnI and DNA mixture contained both protein and target oligonucleotide duplex (i.e. that neither component had crystallized alone). DIBER further indicated that two DNA molecules were present in the asymmetric unit, pointing roughly along the diagonals of the ab (a*b*) plane. The conclusion about two DNA duplexes in the asymmetric unit was also supported by the 2-fold self-rotation function, which clearly showed the grand circles of 2-fold axes of the DNA phosphodiester backbones. The structure was solved by molecular replacement with the help of MolRep program (19). As a model we have used the previously published R.DpnI structure, in which the DNA was only bound to the winged helix domain of the enzyme (16). It was not possible to detect a clear rotation/translation signal with either the complete enzyme or any of the domains in isolation. A plausible solution was obtained with a model of the catalytic domain bound to DNA. The catalytic domain and the DNA were independently superposed on the complex of R.PvuII restriction enzyme (also a blunt end cutter) with its target oligonucleotide duplex [PDB code 1F0O (20)]. With the resulting search model, we still could not obtain a clear rotation signal, but at the translation function stage, one solution stood out (translation function contrast 6.64 versus 3.26 for the next highest peak). Using this solution as a fixed input to a second molecular replacement run, we were then able to orient and position the complex of the winged helix domain and DNA. The correctly placed complete model was characterized by an R-factor of 52.7% (compared to over 55% for all incorrect solutions). A few cycles of manual DNA adjustment and ARP/wARP automatic model building (21) improved the *R*_cryst_ to ∼30% and *R*_free_ to ∼35%. The structure was then refined with the programs COOT (22), REFMAC (23) and CNS (24). The refinement statistics are presented in Supplementary Table S1.

### Cleavage assay

5.5 nM of either non-methylated or methylated pBR322 plasmid DNA substrate (concentration of GATC sites: 121 nM) was used for the cleavage assay. The methylated plasmid was isolated from a Dam+ *E. coli* strain. The concentration of R.DpnI (wild-type enzyme or its variants) was 32.5 nM. The cleavage assay was performed for 2 h at 37°C in 20 μl reaction volume of the standard Tango buffer (Thermo Scientific). The optimal concentration range was determined for the wild-type R.DpnI in the same conditions with a set of enzyme dilutions (concentration range from 32.5 to 0.03 nM) (Supplementary Figure S1).

### Hydrogen/deuterium exchange experiments

Hydrogen/deuterium exchange mass spectrometry (HDXMS) studies were performed as previously described (25), with some modifications. Briefly, to determine the sequence coverage by peptic peptides of R.DpnI protein or its alanine substitution variants, 5 μl of each protein stock (160 μM) was diluted 10-fold by adding 45 μl of H_2_O reaction buffer (20 mM Tris-HCl pH 7.5, 150 mM NaCl). The sample was then acidified by mixing with 10 μl of H_2_O stop buffer (2 M glycine, 4 M guanidine hydrochloride, pH 2.5) and digested on an immobilized pepsin column (Poroszyme Immobilized Pepsin, ABI) with 0.07% formic acid in water as a mobile phase (flow rate 200 μl/min). Digested peptides were passed to the C18 trapping column (Acquity BEH C18 VanGuard Pre-column, Waters) and then directed onto a reverse-phase column (Acquity UPLC BEH C18 column; Waters) with a 6–40% gradient of acetonitrile in 0.1% formic acid at 40 μl/min using nanoACQUITY Binary Solvent Manager. All fluidics, valves and columns were maintained at 0.5°C with HDX Manager, except for the pepsin column that was kept at 13°C inside the temperature-controlled digestion compartment of the HDX Manager. C18 column outlet was coupled directly to the ion source of SYNAPT G2 HDMS (Waters). For protein identification, mass spectra were acquired in MSE mode over the m/z range of 50–2000. Spectrometer parameters were as follows: ESI (electrospray ionization) positive mode, capillary voltage 3 kV, sampling cone voltage 35 V, extraction cone voltage 3 V, source temperature 80°C, desolvation temperature 175°C and desolvation gas flow 800 l/h. Peptides were identified using ProteinLynx Global Server software (PLGS, Waters). The list of identified peptides was passed to the DynamX 2.0 program (Waters).

Hydrogen/deuterium exchange experiments were carried out without DNA (apo) and in the presence of 1 and 2 stoichiometric equivalents of either methylated or non-methylated DNA as a control (DNA strands 5′-ATATGG**GXTC**GTCAGTCAGCG-3′ and 5′-CGCTGACTGAC**GXTC**CCATAT-3′, where X is either 6-methyladenine or adenine). Each protein variant was mixed with 21 bp DNA duplex in the presence of 5 mM Ca^2+^ ions (to obtain 65 μM protein concentration and 65 μM or 130 μM DNA concentration) and allowed to bind for 30 min at room temperature. Then, the experiments were carried out as described above for non-deuterated samples, but H_2_O was replaced with D_2_O. After mixing 5 μl of protein or protein-DNA complex with 45 μl of D_2_O reaction buffer, the exchange reactions were carried out for 10 s at room temperature. The exchange was quenched by reducing pH by adding the reaction mixture to D_2_O stop buffer cooled on ice. The sample was then incubated for 2 min on ice and immediately injected. Spectrometer parameters were the same as described above, but additionally SYNAPT G2 HDMS worked in ion mobility mode.

Two control experiments were carried out to take into account in- and out-exchange artifacts. Briefly, to calculate minimum exchange (IN control), D_2_O reaction buffer was added to D_2_O stop buffer cooled on ice prior to the addition of protein stock, kept for 2 min on ice, and subjected to pepsin digestion and liquid chromatography mass spectrometry (LC-MS) analysis. The deuteration level in an in-exchange experiment was denoted as 0% exchange (*M*_ex_0). For the OUT control, 5 μl of protein stock was mixed with 45 μl of D_2_O reaction buffer, incubated overnight, then mixed with D_2_O stop buffer and analyzed as described above. The deuteration level in an out-exchange control experiment was calculated and denoted as 100% exchange (*M*_ex_100).

### HDXMS data analysis

Deuteration levels for each peptide resulting from the exchange were calculated with DynamX 2.0 software, based on the peptic peptide list obtained from the PLGS program, which was further filtered in the DynamX 2.0 program with the following acceptance criteria: minimum intensity threshold of 1000 and minimum products per amino acids of 0.3. Analyses of the isotopic envelopes after exchange were carried out with the following parameters: retention time deviation ± 15 s, m/z deviation ± 12.5 ppm, drift time deviation ± 2 time bins. Final data were exported to Excel (Microsoft Office) for calculations. Percentage of deuterium uptake was calculated with a formula that takes into consideration the minimum (*M*_ex_0) and maximum exchange (*M*_ex_100) of a given peptide:

\begin{equation*}
D(\% ) = \frac{{\left( {M_{{\rm ex}} - M_{{\rm ex}} 0} \right)}}{{\left( {M_{{\rm ex}} 100 - M_{{\rm ex}} 0} \right)}} \times 100\%.
\end{equation*}

Error bars for percentage of deuteration (%*D*) represent standard deviations of three independent experiments, except for two cases (wt R.DpnI:mDNA 1:2 complex and K229A/R231A R.DpnI variant:mDNA 1:1 complex), for which the error was calculated as a range between duplicates.

## RESULTS

### Crystallization and structure determination

In order to grow crystals of R.DpnI with substrate DNA bound to both the catalytic and winged helix domains, we increased the stoichiometric ratio of DNA to enzyme from the previously used 1:1 to 3:1. We then screened anew for possible crystallization conditions in the presence of Ca^2+^, but not Mg^2+^ ions to promote DNA binding, but not cleavage. This approach led to the identification of a new crystal form in space group *C*222_1_. Crystals diffracted to a resolution of 2.35 Å on a synchrotron beamline and turned out to contain one molecule of R.DpnI together with two duplexes of target DNA in the asymmetric unit. The symmetry-related DNA molecules are stacked in such a manner to create long ‘threads’ extending through the entire crystal (Supplementary Figure S2). We solved the structure by molecular replacement and refined it to crystallographic R-factors *R*_cryst_ = 20.1% and *R*_free_ = 21.9% (Supplementary Table S1).

### Overall structure

**Figure 1.**
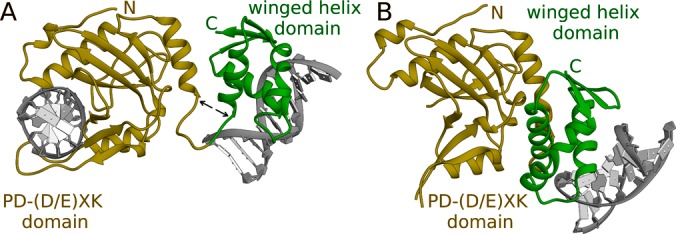
Overall comparison of the R.DpnI structures. The co-crystal structures of R.DpnI with either two DNA duplexes (**A**) or one DNA duplex (16) (**B**) per R.DpnI molecule with their catalytic domains oriented in the same way. PD-(D/E)XK and winged helix domains of the protein are shown in gold and green, respectively, and the DNA is in gray. The termini of the helices that are unwound in (A) but not in (B) are marked with black arrows..

Both DNA duplexes in the crystal are canonically Watson–Crick paired. This applies also to the methylated bases. As expected, the R.DpnI catalytic PD-(D/E)XK and winged helix domains (residues 1-182 and 183-254, respectively) from the current and previously solved structure superimpose well (main chain RMSD of 3.0 Å for residues 1-174, 1.2 Å for the 60% at the core of the catalytic domain; and 0.3 Å for residues 187-254). However, the relative domain orientations differ drastically between the two structures [according to the DynDom server (27) the change can be described as a 75°–80° rotation] (Figure 1). The new arrangement requires unwinding of the first helix of the winged helix domain (by ∼1.5 turn) and the last helix of the catalytic domain (by about a turn). Both effects contribute to the lengthening of the linker region. Binding of the DNA duplex to the winged helix domain is barely influenced by these changes. All interactions with the target sequence are preserved and only minor and probably insignificant variations of hydrogen-bond lengths are observed. Binding of DNA makes the catalytic domain ‘lock in’ on its target. Some parts of the protein that could not be reliably traced in the former structure are now well defined in the electron density. The loops of the PD-(D/E)XK domain get ordered and embrace the DNA duplex from major and minor grooves (Figure 1).

### The active site

The catalytic region of R.DpnI is disordered in the absence of the substrate, but folds upon DNA binding and forms a standard PD-(D/E)XK active site with two binding sites for divalent cations. Active R.DpnI would have Mg^2+^ ions bound in these positions, but in the crystal these sites are instead occupied by Ca^2+^ or Na^+^ ions from the buffer. Based on the anomalous X-ray diffraction signal, we suspect that one of the sites (site 1) is nearly fully occupied by a Ca^2+^ ion, while the other site (site 2) may be occupied by a mixture of Ca^2+^ and Na^+^ ions (Supplementary Figure S3).

The site 1 Ca^2+^ ion is hexacoordinated by the side chains of Asp53 (the ‘bridging’ ligand) and Glu64, as well as by the main chain carbonyl oxygen atom of Leu65, the proS (OP2) non-bridging oxygen of the ‘scissile’ phosphate, and two water molecules. One of them is positioned roughly in line with the scissile P-O3′ bond and located within hydrogen-bonding distance of the catalytic Lys66. This water molecule would be incorporated into the substrate if the reaction proceeded. In the crystal, this does not happen, as evidenced by the length of the scissile bond and its continuous density. The water phosphorus distance is too large (3.4 Å), due to binding of the ‘wrong’ metals in the active site. The site 2 ion is also hexacoordinated by the side chains of the bridging Asp53 residue, proS and 3′ oxygen atoms of the ‘scissile’ phosphate and three water molecules. If the reaction took place, the contact of this metal cation to the leaving group O3′ atom would promote its departure. We note that our structure-based assignment of residue roles is consistent with the earlier bioinformatic prediction and site-directed mutagenesis experiments (16).

### Sequence recognition by the catalytic domain

The R.DpnI Gm6ATC target sequence is palindromic, but like the winged helix domain, the catalytic domain does not exploit this symmetry. Bases of the proximal (poised for cleavage) and distal (not poised for cleavage) strands are recognized differently. Two regions of the R.DpnI PD-(D/E)XK domain mediate most interactions with the DNA. A three-stranded antiparallel β-sheet with a long loop (residues 75-80 and 123-144) wedges into the major groove of the target DNA. The most N-terminal α-helix of the enzyme (residues 16-31, preceded by an unstructured fragment with a single 3_10_ helix turn) contacts DNA from the minor groove side. As R.DpnI monomer contacts a full recognition site rather than only a half-site, dimerization of the enzyme on its palindromic DNA target would result in major clashes and is therefore very unlikely.

### Readout of the outer G:C pairs

**Figure 2.**
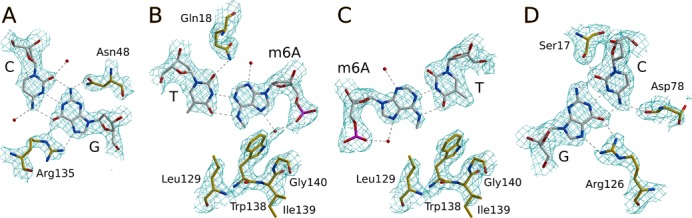
Sequence recognition by the R.DpnI PD-(D/E)XK domain. Amino acids and DNA nucleotides are shown in all-atom representation. The composite omit map was contoured at 1.2σ. Panels (**A–D**) show the Gm6ATC bases of the proximal strand ordered as in the recognition sequence together with paired bases of the distal strand and interacting amino acids.

The G:C pairs of the Gm6ATC target sequence hydrogen bond extensively with R.DpnI (Figure 2 and Supplementary Figure S4). The proximal guanine is hydrogen bonded via its O6 and N7 atoms with the Nϵ and Nζ atoms of Arg135 (which also forms part of the hydrophobic pocket for the methyl groups) and via its N2 atom with the Oδ atom of Asn48 (Figure 2A). The distal guanine accepts two hydrogen bonds to its O6 and N7 atoms from the Arg126 guanidino group. The complementary cytosine donates a hydrogen bond from its N4 to the main chain carbonyl of Asp78 of the first β-strand in the β-sheet. On the minor groove side this C:G base pair is recognized by a single hydrogen bond from the Ser17 Oγ of the N-terminal α-helix to the cytosine O2 atom (Figure 2D). We also observe two additional hydrogen bonds between flanking cytosines on both sides of the target sequence and Asn48 and Asn77, which according to the biochemical data do not contribute to sequence specificity (16,28).

### Readout of the inner m6A:T pairs and methylation specificity

**Figure 3.**
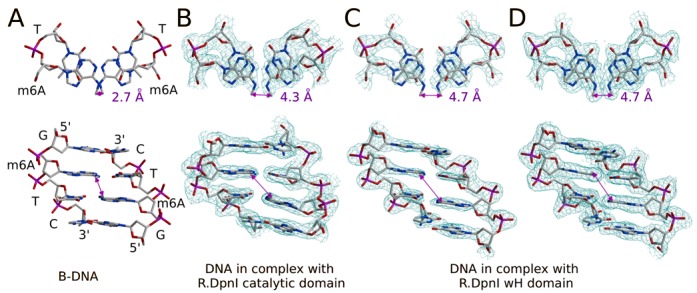
Methyl-methyl distance and DNA distortions in R.DpnI–DNA complex. The carbon-carbon distance between the methyl groups of m6A residues in the Gm6ATC sequence context was measured for idealized B-DNA (**A**), DNA in complex with the R.DpnI catalytic domain (**B**) and DNA in complex with the winged helix domain (**C, D**). Due to slightly better resolution, the density for the winged helix domain was clearer in the previous structure (16) (D), but the model is essentially identical to the current one (C). Both composite omit maps were contoured at 1.2σ.

Both 6-methyladenines have their methyl groups roughly located in the plane of the bases, presumably to preserve the partial double bond character of the C6-N6 bond (i.e. the conjugation of the nitrogen lone pair with the aromatic system of the purine ring). The two possible conformations of the base have been termed ‘*cis*’ and ‘*trans*’ (methyl group pointing toward the Watson-Crick and Hoogsteen edge, respectively) (29). The electron density of the DNA bound to the catalytic domain is consistent with the *trans* conformation (Figure 3). In the present structure, the DNA bound to winged helix domain is not very well ordered, and the density for the m6A methyl groups is poor. However, the much clearer density from the structure of R.DpnI with DNA bound only to the winged helix domain confirms the *trans* conformation for the methyl groups (Figure 3D) (16).

**Figure 4.**
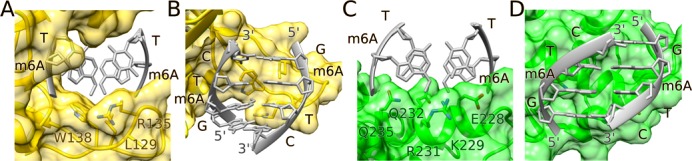
Recognition of the 6-methyladenines by R.DpnI. Interaction of the m6A bases with the R.DpnI catalytic (**A, B**) and winged helix (**C, D**) domains. The methyl groups are in contact with a long loop connecting adjacent antiparallel β-strands in the case of the catalytic domain and an α-helix in the case of the winged helix domain. For clarity, a part of protein was omitted in panel (B) and parts of DNA in all panels.

There is only a single hydrogen bond between the R.DpnI catalytic domain and the two central base pairs of its target sequence, which is donated by the Gln18 Nϵ atom to the O2 atom of the distal thymine (Figure 2B and Supplementary Figure S4). As G:C pairs would have an amino group in the central minor groove position clashing with the Gln18 Nϵ (which is in turn held in place by other hydrogen-bonding interactions), this contact likely contributes to sequence selectivity. All other polar interactions with the bases of the m6A:T pairs are solvent mediated (Figure 2B and C). The main van der Waals contacts between R.DpnI and the inner m6A:T pairs are made by the m6A methyl groups. As the methyl groups are very close to each other, they are bound together in one cleft of R.DpnI catalytic domain (Figure 4 and Supplementary Figure S5). The hydrophobic pocket is formed by Leu129, Arg135 and Trp138, which are all located in the long loop that gets ordered upon DNA binding. Trp138 plays a key role in the recognition of m6A methylation. It simultaneously interacts with both methyl groups: its pyrrole ring is in van der Waals contact with the m6A of the proximal DNA strand and its benzene ring with the m6A of the distal strand. The conformation of Trp138 side chain is stabilized by packing against Gly140 (particularly the NH group, glycine in this position is required because the Cβ atom of any other amino acid would clash with the indole ring of Trp138) and by a solvent-mediated interaction of its Nϵ atom with the m6A phosphate (Figures 2 and 4 and Supplementary Figure S4).

### Site-directed mutagenesis of the methyl binding cleft

**Figure 5.**
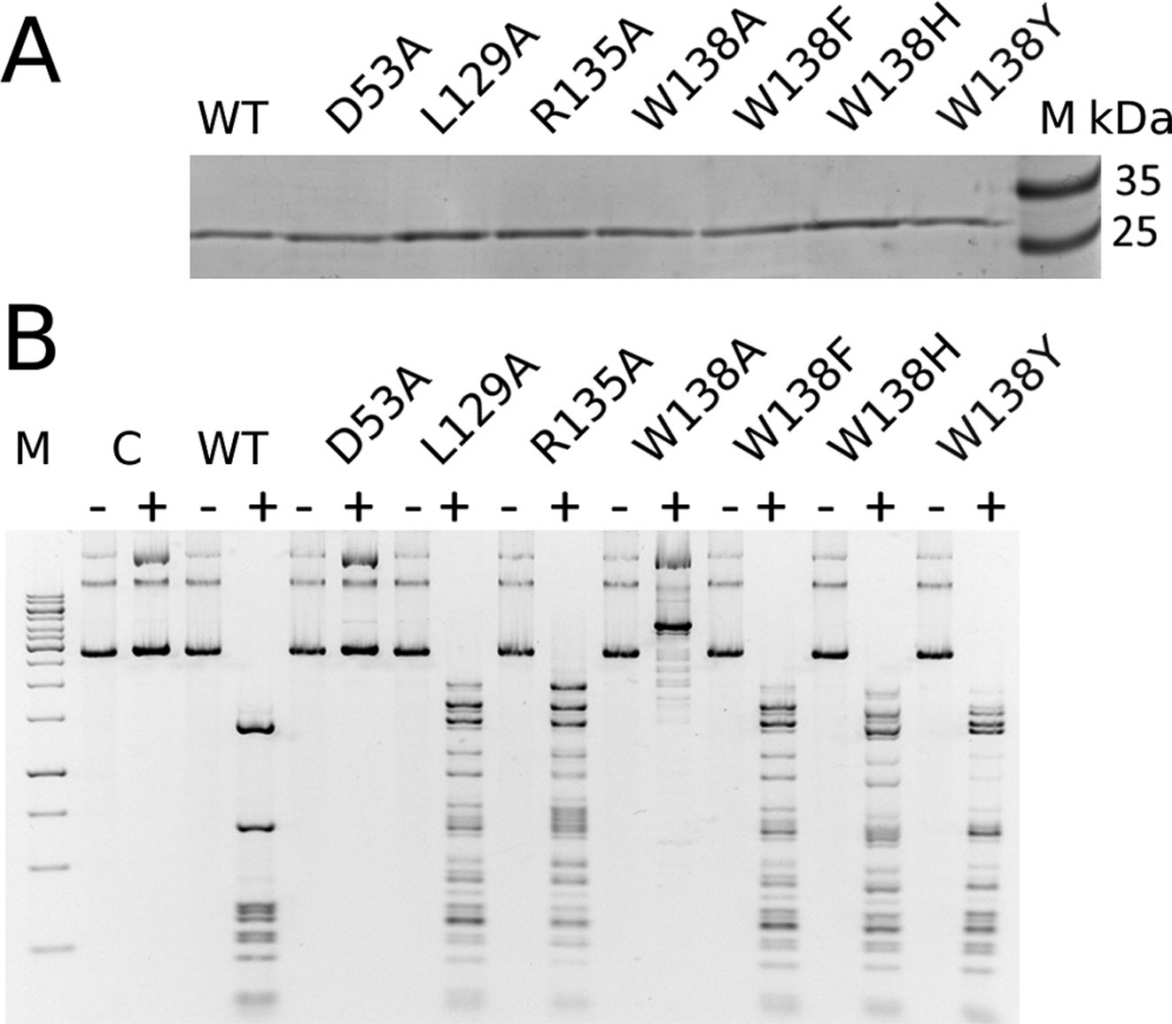
Activity of R.DpnI variants. (**A**) R.DpnI and its variants were analyzed for protein purity by Coomassie-stained SDS-PAGE (1 μg per lane). (**B**) The catalytic activity of wild-type R.DpnI and its variants was tested against either non-methylated or Dam-methylated pBR322 plasmid as stated in the Materials and Methods section. M: molecular mass marker; C: non-cleaved substrate; WT: wild-type R.DpnI; ‘−’: non-methylated DNA; ‘+’: Dam-methylated DNA.

The contribution of residues predicted by the crystal structure to be involved in methyl binding was explored using previously described (16) as well as newly generated R.DpnI variants. Protein activity was tested against non-methylated and Dam-methylated pBR322 plasmid in conditions that require multiple turnover (∼4-fold excess of target sites over enzyme molecules) (Figure 5). An active site substitution (D53A) was used as a control. None of the mutants displayed activity against non-methylated substrate. The L129A and R135A variants retained partial activity against the Gm6ATC target sequence containing DNA. The latter is remarkable, because replacement of the arginine with an alanine abolishes not only methyl group interactions but also two hydrogen bonds with a target sequence guanine. We have therefore tested the R135A variant activity with the help of the Dam-methylated pBR322 plasmid treated with Hia5 methyltransferase that introduces 6mA modification in a broad sequence range (30). However, the R135A variant did not show a clear signature of relaxed specificity (Supplementary Figure S6).

Due to its extensive interactions with the methyl groups, Trp138 was replaced not only with alanine but also with phenylalanine, histidine and tyrosine. The alanine variant almost completely lost activity. The other variants retained partial activity, which is in agreement with the presence of histidine and tyrosine in a number of R.DpnI homologs (predominantly from various *Neisseria* strains). Taken together, the data suggest that R.DpnI exploits not only favorable direct contracts with the methyl groups for its methylation specificity. The enzyme also ‘senses’ the methyl groups indirectly, either via solvation effects like other methyl-specific proteins or via effects that the methyl groups have on DNA conformation.

### Hydrogen/deuterium exchange experiments and ‘crosstalk’ between R.DpnI domains

We have previously shown that separate sequence and methylation specificity of the R.DpnI domains suggests a ‘double’ readout, which should enhance methylation specificity. We have further demonstrated that the presence of a second target site in a substrate substantially increases the enzyme efficiency (16). However, it was not clear from the previous data whether enhanced specificity was simply an avidity effect, or whether the effector domain could somehow ‘communicate’ the binding of cognate DNA to the catalytic domain.

In order to address this question, we took advantage of the earlier identification of two amino acid exchanges in the winged helix domain (K229A, R231A), which separately impair the activity of R.DpnI and created a new double substituted variant (K229A/R231A). We then compared binding of a 21-mer DNA oligoduplex (sequence in the Materials and Methods section) by wild-type R.DpnI and its R231A and K229A/R231A variants by mass spectrometry-monitored hydrogen/deuterium exchange. Experiments were carried out with no DNA, or with either non-methylated or methylated target DNA in either 1:1 or 2:1 stoichiometric ratio.

**Figure 6.**
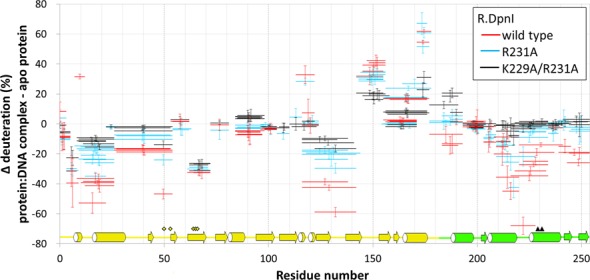
Change of deuteration upon addition of methylated DNA. Two molar equivalents of DNA and 10 s exchange time were used. Negative values indicate protection from hydrogen/deuterium exchange upon DNA binding and positive values indicate destabilization of hydrogen-bonding network of a given peptide. R.DpnI secondary structure is indicated (catalytic domain in yellow and winged helix domain in green). Active site residues are marked by yellow diamonds. Positions 229 and 231 that were mutated to weaken DNA binding are indicated by black triangles.

The obtained data indicated high sequence coverage and demonstrated that non-methylated DNA had little (less than 20% change in deuteration) effect on the deuterium uptake of R.DpnI or its variants, regardless of whether DNA was present in 1:1 or 2:1 stoichiometric ratio to the protein (Supplementary Figures S7, S8A and B). In contrast, methylated DNA shielded both the catalytic and winged helix domains of wt R.DpnI protein from D_2_O exchange (Figure 6 and Supplementary Figures S7 and S8C).

The shielding effect of methylated DNA was more pronounced when the DNA was present in 2:1 ratio (Figure 6), but some protection was also observed for 1:1 molar ratio (Supplementary Figure S8C). Increased percentage of deuteration in the presence of DNA was observed close to the linker between the domains and in the preceding β-strand (around residues 150 and 175). Regions of reduced deuteration map roughly to the DNA-binding sites (Supplementary Figure S9). However, the agreement is not perfect, due to the limited amino acid resolution of hydrogen/deuterium exchange experiments (which in turn is due to the length of analyzed peptides).

R.DpnI variants were less shielded than the wild-type protein in the winged helix domain, confirming the earlier data that the introduced mutations compromised DNA binding by this domain. As expected, the domain with double alanine substitution was even less sensitive to the presence of methylated DNA than with the single one. Information about possible crosstalk between R.DpnI domains is contained in the effects of mutations in the winged helix domain onto the DNA-binding properties of the catalytic domain. If the domains were fully independent, then amino acid substitutions in one of them should not affect the other. This is not what is observed. Instead, the experiments indicate that particularly the K229A/R231A variant of R.DpnI is severely compromised in binding of DNA to the catalytic domain, even though this domain is exactly the same as in the wild-type protein.

## DISCUSSION

### Recognition of the two methyl groups in a single cleft of the enzyme

In fully methylated Gm6ATC sequence, the methyl groups are in *trans* and so close that R.DpnI binds them as a single hydrophobic entity rather than separately (Figure 4 and Supplementary Figure S5). The contribution of the direct interactions between DNA and the R.DpnI catalytic domain is demonstrated by the loss or impairment of its enzymatic activity when the residues of the methyl binding cleft are mutated (Figure 5). The contact area to either the entire m6A bases or just the methyl groups is greatest for Trp138, intermediate for Arg135 (which also hydrogen bonds a guanine of the recognition sequence) and smallest for Leu129. This is in qualitative agreement with the results of digestion experiments that show that the W138A exchange has more severe effects on substrate turnover than the other amino acid substitutions. The mutagenesis experiments show considerable robustness of R.DpnI activity and methylation specificity to substitutions of residues contributing to the methyl binding cleft, suggesting that methylation specificity is not only due to direct interactions.

### Methyl group proximity and resulting constraints on DNA conformation in solution

**Figure 7.**
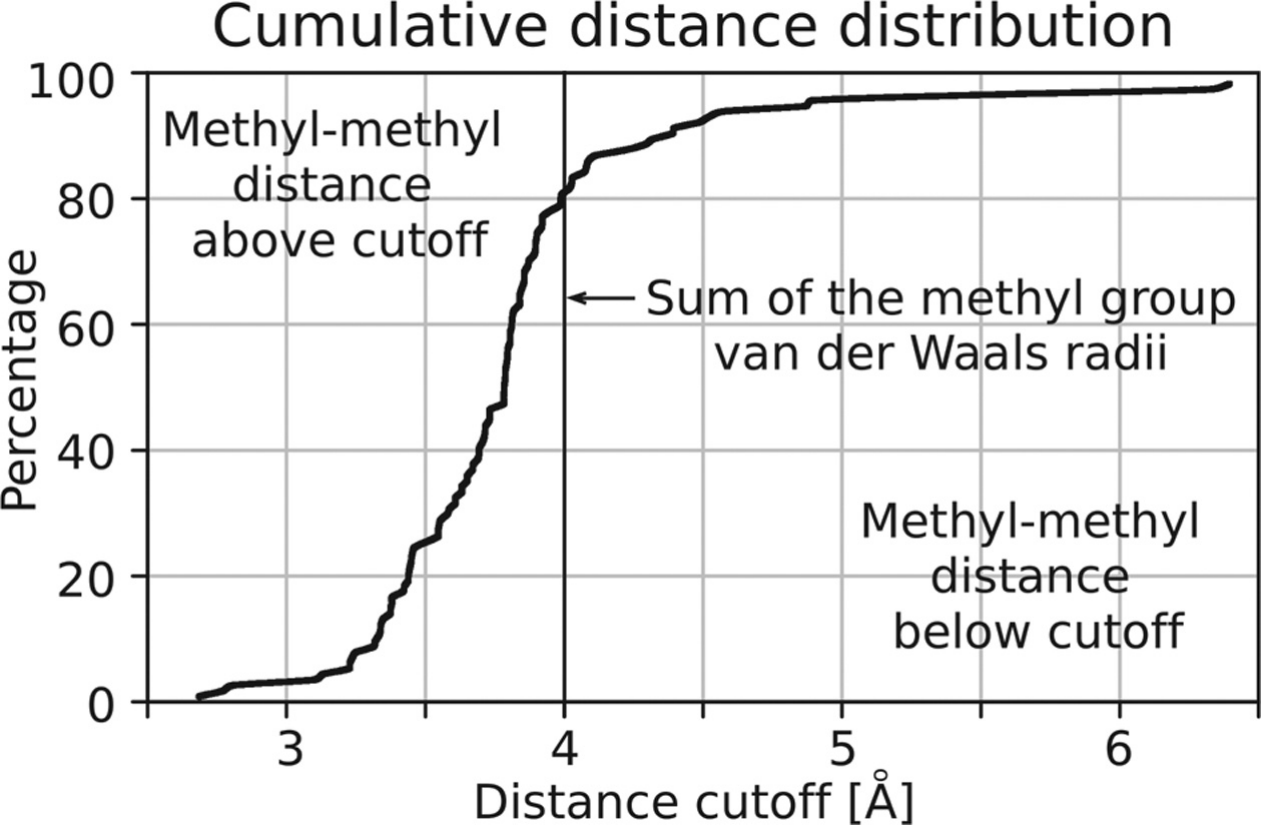
Methyl-methyl distances. Cumulative distribution for the carbon-carbon distance between methyl groups modeled in the *trans*conformation on the two adenines of 114 structures in the PDB with the GATC sequence (and resolution ≤2.5 Å, R-factor ≤25%). For around 80% of all models, the distance between the methyl groups is below the 4.0 Å sum of methyl group van der Waals radii.

In fully methylated DNA, the two methyl groups on the adenines are in adjacent base pairs close to the central minor groove. When methyl groups are grafted on ideal B-DNA with the GATC sequence [generated with default parameters by the 3DNA program (31)], then the carbon-carbon distance between these methyl groups is 2.7 Å. Due to propeller twist, this value is smaller than the distance between DNA base pairs. More importantly, it is also smaller than the sum of van der Waals radii of two methyl groups (2.0 Å + 2.0 Å = 4.0 Å) (32). Therefore, we conclude that methyl group proximity must substantially constrain the conformational freedom of DNA in solution. In order to exclude that the clash of methyl groups was a particular feature of ideal B-DNA, we retrieved the structures of all DNA duplexes with the GATC sequence (at least 2.5 Å resolution, crystallographic R-factor below 25%) from the PDB. As for B-DNA, *trans* methyl groups were grafted on the adenines. In ∼80% of the cases, the distance between the methyl groups is smaller than the 4.0 Å threshold value for a clash (Figure 7), indicating that these DNA conformations could not be adopted by methylated DNA in solution. We also noted that stacking effects of the methyl groups against each other and adjacent DNA bases might further constrain the flexibility of methylated DNA in solution (Supplementary Figure S10).

### Avoidance of a methyl-methyl clash in the R.DpnI DNA co-crystal structures

The DNA molecules that are bound to the catalytic and winged helix domains of R.DpnI are deformed relative to the ideal B-DNA so that the m6A methyl groups do not clash (Figure 3). The distance between the methyl groups of the palindromic m6A:T dinucleotide pair can be enlarged by a reduction of the propeller twist (paired bases more fully in one plane), by an increase of the roll angle to open up the major grove, by an opening of the base pair and also by an increase of distance between base pairs. In the crystal structure, several of these parameters contribute to bring the methyl groups apart. The DNA distortions have largely local effects and do not introduce major kinks or bends in the target site (Figure 3 and Supplementary Table S2). This observation is consistent with earlier conclusions from experiments about DNA structure in solution, which have shown that adenine methylation kinks/bends DNA in some sequence contexts (e.g. GAm6ATTC, the product of M.EcoRI methylation), but not others including Gm6ATC (the Dam methylation product and R.DpnI target) (33).

### R.DpnI is likely to sense methyl group dependent changes in DNA conformation

R.DpnI could sense methyl groups in its substrates by their effect on DNA conformation. At least two different not mutually exclusive scenarios are conceivable. The first scenario focuses on the set of configurational states that is accessible to non-methylated, but not to methylated DNA in solution. Assuming that both are much constrained in their conformational freedom upon protein binding, one might expect a lower ‘entropic’ price for binding of methylated DNA to R.DpnI. The second possible scenario focuses on the influence of the methyl groups on pre-forming a DNA conformation in solution that fits the DNA-binding sites in the catalytic and winged helix domains. According to this latter scenario, the tighter binding of methylated compared to non-methylated DNA need not be entropically driven.

Proximity of m6A methyl groups in Watson-Crick paired DNA duplexes is unique for the one nucleotide stagger found in the R.DpnI target sequence (Supplementary Table S3). A similarly close approach is not possible for the methyl groups of 5-methylcytosines, since these are located on the periphery of the major grove. The clash between the methyl groups of 4-methylcytosines in B-DNA is also less severe than that observed for 6-methyladenines. Hence, the conformational effects described here are unlikely to occur for adenine methylation with a different stagger or for any form of cytosine methylation.

### Solvation/desolvation effects in methyl-specific recognition

Desolvation effects have been reported to contribute to 5-methylcytosine specificity of zinc finger protein Zfp57 (3) and methyl CpG binding protein MeCP2 (4). As desolvation of a hydrophobic methyl group should generally be more favorable than desolvation of a hydrogen atom/proton, the reference to solvation effects to explain methyl recognition is not surprising. In the case of the R.DpnI substrate complex, the situation is atypical, because the two methyl groups of fully methylated Gm6ATC motif are jointly accommodated in a cleft of R.DpnI (of either its winged helix or catalytic domain). The proximity of the methyl groups in DNA implies that their partial desolvation must have occurred already in the absence of protein. Nevertheless, the pair of methyl groups is sufficiently surrounded by protein amino acids in the complex at least in the catalytic domain that we suspect that solvation/desolvation effects also play a role for R.DpnI methylation specificity.

### Cooperation between the R.DpnI domains

The hydrogen/deuterium exchange experiments indicate that the mutations in the R.DpnI winged helix domain can severely affect the binding of target DNA by the catalytic domain. This suggests communication between the domains, which we expect to enhance R.DpnI specificity. We note that the indirect effects of one domain onto the other cannot be attributed to simple titration. Even for the 1:1 stoichiometric ratio of protein and DNA, failure of the winged helix domain to bind target oligoduplex should increase the amount of DNA in solution available for the catalytic domain, and thus increase, rather than decrease its protection. In the 2:1 stoichiometry experiment, the same argument applies, but there is now sufficient DNA in solution that even if DNA was bound to the winged helix domain, there would always be enough DNA to shield the catalytic domain. We conclude from these data that there is crosstalk between the two parts of the protein, which affects the binding affinity of the catalytic domain for DNA, and might also affect the rate of catalysis, although this cannot be concluded from the data. We believe that the crosstalk further enhances the methylation specificity of the individual domains, which we attribute to a combination of direct interactions, solvation/desolvation effects and effects of DNA methylation on its conformation mediated by the proximity of the methyl groups in the R.DpnI substrate.

## ACCESSION NUMBER

The atomic coordinates and structure factors of the reported R.DpnI DNA complex have been deposited in PDB under the 4KYW accession code.

## SUPPLEMENTARY DATA

Supplementary Data are available at NAR Online.

SUPPLEMENTARY DATA
