# Supplementary material for: Structural basis of the methylation specificity of R.DpnI
Source: Nucleic Acids Res. 2014 Jun 25;42(13):8745–54. doi: 10.1093/nar/gku546 (PMC4117772; doi:10.1093/nar/gku546)
Supplement: SUPPLEMENTARY DATA [file supp_42_13_8745__index.html]

Structural basis of the methylation specificity of R.DpnI — SUPPLEMENTARY DATA 

# Structural basis of the methylation specificity of R.DpnI

## SUPPLEMENTARY DATA

**Files in this Data Supplement:**

- SUPPLEMENTARY DATA
