# Supplementary material for: Structural basis of the methylation specificity of R.DpnI
Source: Nucleic Acids Res. 2014 Jun 25;42(13):8745–54. doi: 10.1093/nar/gku546 (PMC4117772; doi:10.1093/nar/gku546)
Supplement: SUPPLEMENTARY DATA [file supp_gku546_nar-01663-h-2013-File009.pdf]

Supplementary Material for:

Structural basis of the methylation specificity of R.DpnI

Karolina Mierzejewska[1,*], Wojciech Siwek[1,*,$], Honorata Czapinska[1,*], Magdalena Kaus-Drobek[2], Monika Radlinska[3], Krzysztof Skowronek[1], Janusz M. Bujnicki[1,4], Michal Dadlez[2], Matthias Bochtler[1,2,$]

[1]*International Institute of Molecular and Cell Biology, Trojdena 4, 02-109 Warsaw, Poland*

[2]*Institute of Biochemistry and Biophysics PAS, Pawinskiego 5a, 02-106 Warsaw, Poland*

[3]*Institute of Microbiology, Faculty of Biology, University of Warsaw, Miecznikowa 1, 02-096 Warsaw, Poland*

[4]*Institute of Molecular Biology and Biotechnology, Adam Mickiewicz University, Umultowska 89, 61-614 Poznan, Poland*

*Equal contribution

[$]Corresponding authors:

Tel: +48225970730; +48225970732

e-mail: wsiwek@iimcb.gov.pl, mbochtler@iimcb.gov.pl

Supplementary Tables:

Table S1. Data collection and refinement statistics.

Data collection statistics	
Space group	$C\ 2\ 2\ 2_1$
a (Å)	95.7
b (Å)	101.0
c (Å)	114.8
Resolution range (Å)	50 - 2.35
Total reflections	149639
Unique reflections	23502
Completeness (%) (last shell)	99.7 (99.9)
I/σ (last shell)	20.5 (4.1)
R(sym) (%) (last shell)	4.9 (43.9)
B(iso) from Wilson ($Å^2$)	58.0
Refinement statistics	
Protein atoms excluding H	2096*
DNA atoms excluding H	812
Solvent molecules	97
R_{cryst} (%)	20.1
R_{free} (%)	21.9
RMSD bond lengths (Å)	0.011
RMSD angles (°)	1.3
Ramachandran favored region (%)	96.5
Ramachandran allowed region (%)	100.0
Ramachandran disallowed region (%)	0.0

* Alternative conformations counted separately.

Table S2. DNA distortion parameters according to the 3DNA software. The absolute values are provided for ideal B-DNA and the Δ values with respect to it for the actual R.DpnI bound DNA molecules. The parameters marked in grey/bold are likely to influence the methyl-methyl distance between the neighboring m6A residues in the R.DpnI DNA complex structures. In some cases only the parameter for the "second" T:m6A pair affects the methyl-methyl distance.

	**B-DNA**		**Difference between parameters for R.DpnI bound DNA and ideal B-DNA with the same sequence (Δ)**							
			**this structure cat. domain**		**this structure wH domain**		**4ESJ mol1 wH domain**		**4ESJ mol2 wH domain**	
	**A-T**	**T-A**	**m6A-T**	**T-m6A**	**m6A-T**	**T-m6A**	**m6A-T**	**T-m6A**	**m6A-T**	**T-m6A**
Shear [Å]	0.03	-0.03	0.36	-0.03	0.32	-0.19	0.1	-0.12	0.05	0.07
Stretch [Å]	-0.1	-0.1	0.03	-0.16	-0.08	-0.04	-0.01	0.01	0.03	-0.05
Stagger [Å]	0.09	0.09	-0.38	-0.22	-0.34	-0.07	-0.17	0.08	0.11	-0.01
Buckle [°]	-0.04	0.04	-18.59	-9.8	-2.63	-3.94	-6.32	-1.95	5.23	-1.15
**Propeller-Twist [°]**	**-15.13**	**-15.13**	**1.01**	**14.23**	**12.79**	**11.36**	**11.96**	**7.28**	**9.57**	**10.99**
**Opening [°]**	**-1.88**	**-1.88**	**10.99**	**10.79**	**6.68**	**6.04**	**6.36**	**5.65**	**10.73**	**9.43**
Shift [Å]	-0.03	0	0.21	-0.43	0.36	-0.37	0.31	0.15	0.37	-0.41
**Slide [Å]**	0.5	**0.44**	-1.95	**-1.38**	-1.44	**-1.78**	-1.53	**-1.71**	-1.21	**-1.49**
Rise [Å]	3.37	3.35	0.27	-0.18	0.28	0.14	0.29	-0.05	-0.1	0.32
Tilt [°]	-0.01	0	1.89	1.7	0.77	-1.52	3.96	-1.31	-3.24	-0.96
**Roll [°]**	1.72	**1.7**	6.97	**2.58**	-8.15	**-4.92**	-8.25	**-6.8**	-3.06	**-10.8**
**Twist [°]**	37.13	**35.67**	-0.3	**-4.89**	-4.28	**-8.1**	2.1	**-7.42**	-2.88	**-4.44**

Table S3: Carbon-carbon distance between methyl groups modeled on bases in bold: on adenines in N6 and cytosines in N4 position in a conformation pointing towards the Hoogsteen edge and thus not disrupting Watson-Crick base pairing; and on cytosines in C5 position.

**6-methyladenine**					
sequence context	$CH_3 - CH_3$ distance [Å]	sequence context	$CH_3 - CH_3$ distance [Å]	sequence context	$CH_3 - CH_3$ distance [Å]
5′-**A**T-3′<br>3′-T**A**-5′	2.7	5′-T**A**-3′<br>3′-**A**T-5′	6.8	5′-**AA**-3′<br>3′-TT-5′	4.1
5′-**A**XT-3′<br>3′-TX**A**-5′	5.9	5′-TX**A**-3′<br>3′-**A**XT-5′	10.1	5′-**A**X**A**-3′<br>3′-TXT-5′	8.0
5′-**A**XXT-3′<br>3′-TXX**A**-5′	9.8	5′-TXX**A**-3′<br>3′-**A**XXT-5′	13.3	5′-**A**XX**A**-3′<br>3′-TXXT-5′	11.8
**4-methylcytosine**					
5′-**C**G-3′<br>3′-G**C**-5′	3.5	5′-G**C**-3′<br>3′-**C**G-5′	8.2	5′-**CC**-3′<br>3′-GG-5′	4.4
5′-**C**XG-3′<br>3′-GX**C**-5′	6.0	5′-GX**C**-3′<br>3′-**C**XG-5′	11.3	5′-**C**X**C**-3′<br>3′-GXG-5′	8.6
5′-**C**XXG-3′<br>3′-GXX**C**-5′	10.0	5′-GXX**C**-3′<br>3′-**C**XXG-5′	14.2	5′-**C**XX**C**-3′<br>3′-GXXG-5′	12.5
**5-methylcytosine**					
5′-**C**G-3′<br>3′-G**C**-5′	7.6	5′-G**C**-3′<br>3′-**C**G-5′	11.9	5′-**CC**-3′<br>3′-GG-5′	4.8
5′-**C**XG-3′<br>3′-GX**C**-5′	6.9	5′-GX**C**-3′<br>3′-**C**XG-5′	13.8	5′-**C**X**C**-3′<br>3′-GXG-5′	9.5
5′-**C**XXG-3′<br>3′-GXX**C**-5′	8.9	5′-GXX**C**-3′<br>3′-**C**XXG-5′	15.5	5′-**C**XX**C**-3′<br>3′-GXXG-5′	13.6

Supplementary Figures:

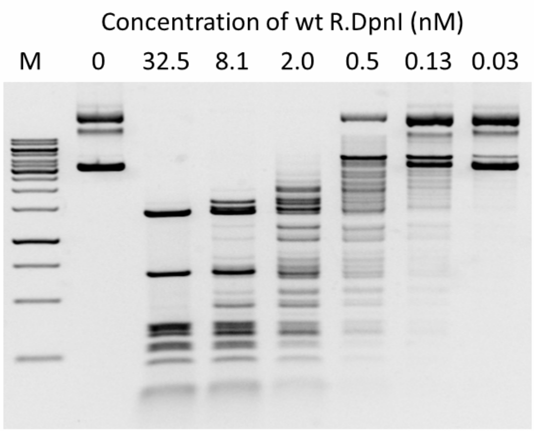

Figure S1: Concentration dependent activity of wild type R.DpnI. Cleavage assay was performed as stated in Materials and Methods, with 5.5 nM Dam methylated pBR322 plasmid DNA substrate (concentration of Gm6ATC sites: 121 nM) and decreasing concentrations of the enzyme.

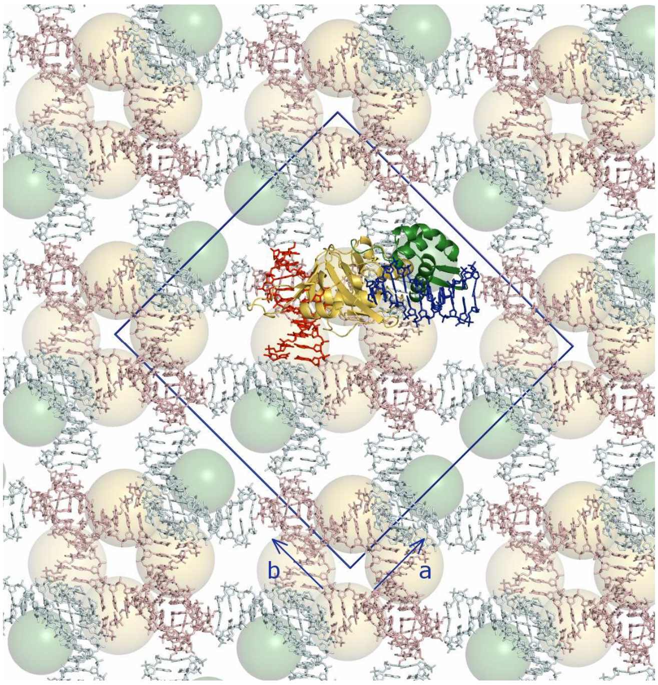

Figure S2: Packing of the R.DpnI crystal. The asymmetric unit contains one molecule of R.DpnI (yellow catalytic and green winged helix domain in ribbon representation) and two oligoduplexes (shown in red and blue in stick representation). For all other molecules in the crystal, the DNA is shaded, and the protein domains are represented by balls for clarity. The number of winged helix domains appears twice smaller than the number of catalytic domains because some of the former are eclipsed in the presented view. The blue lines indicate the crystallographic unit cell (ab plane). Please note that the DNA molecules bound to winged helix and catalytic domains alternate in the DNA “rods” running throughout the crystal.

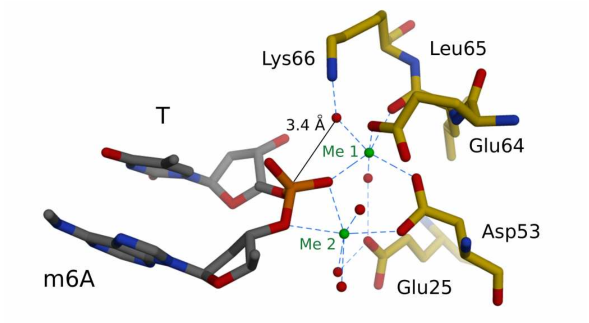

Figure S3: Precleavage conformation of the R.DpnI active site. Selected residues are shown in all-atom representation, water molecules as red spheres and the site 1 and 2 metal ions as green spheres. The coordination of the metal ions and selected hydrogen bonds are shown by dashed blue lines. The nucleophilic water molecule is coordinated by Lys66 and positioned approximately in line with the scissile phosphoester bond (continuous black line).

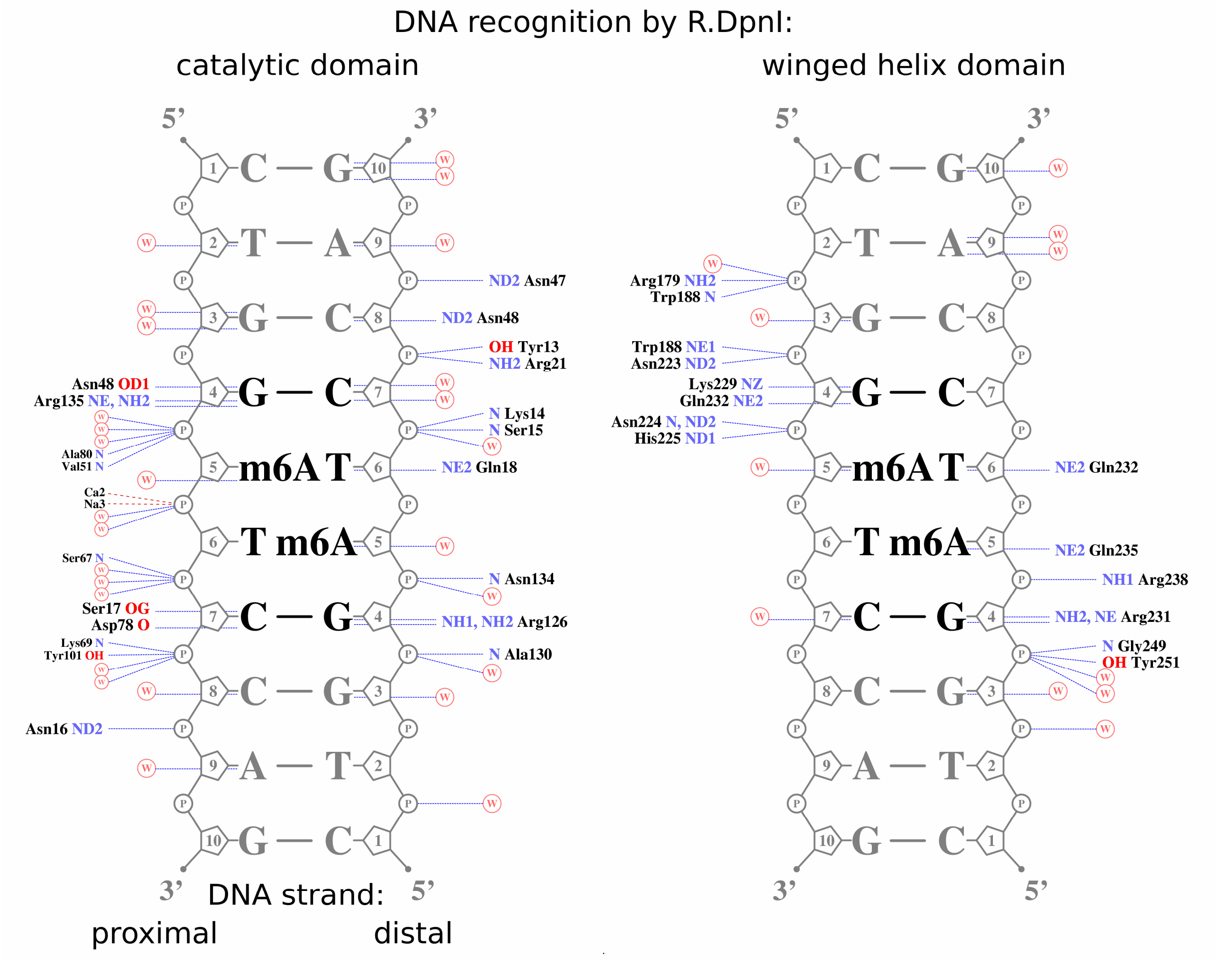

Figure S4: Schematic representation of interactions of the fully methylated DNA with the catalytic (left) and winged helix (right) domains of R.DpnI. The hydrogen bond between one of the flanking cytosines and Asn77 is not shown because of poor geometry.

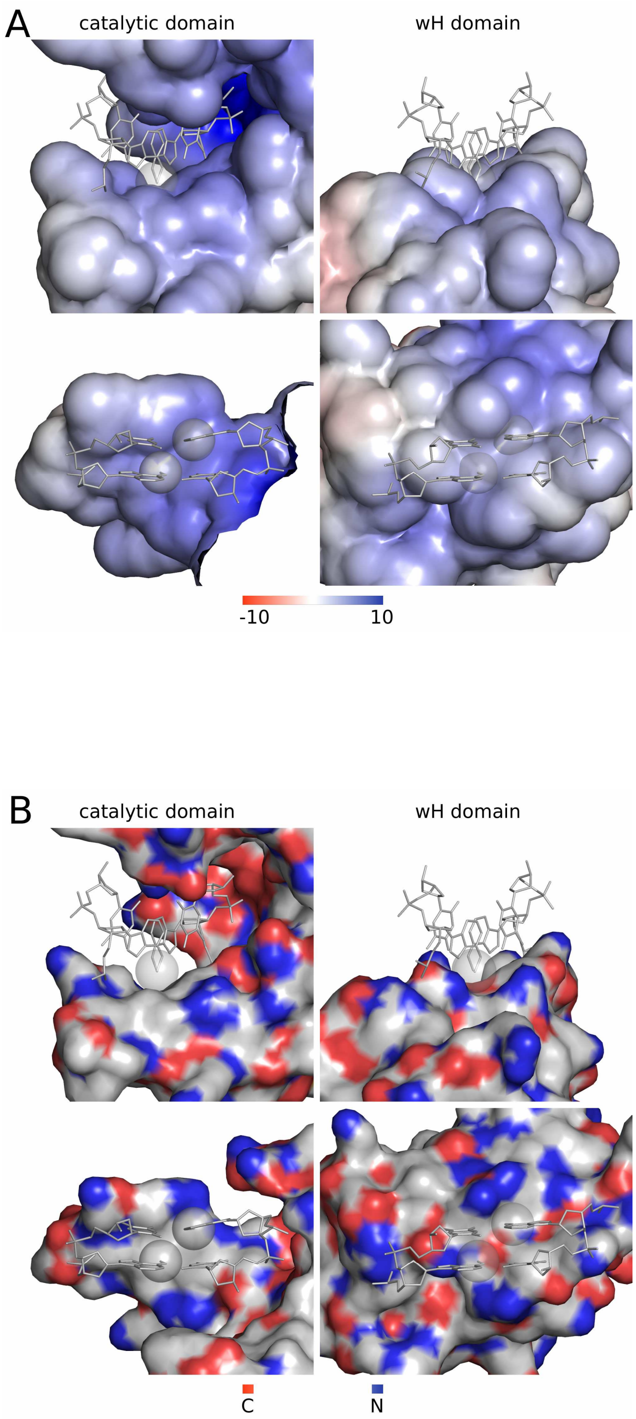

Figure. S5 Methyl binding clefts of R.DpnI domains (for the legend see next page).

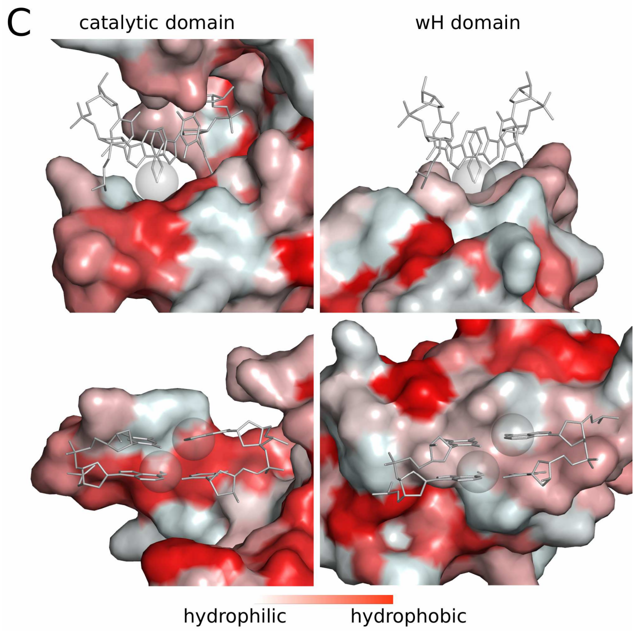

Figure S5: Methyl binding clefts of R.DpnI domains. (A) The R.DpnI solvent excluded surface was colored according to the electrostatic potential (in units of kT/e) calculated with the DelPhi program (Li et al. BMC Biophysics 2012, 5:9). (B) The R.DpnI solvent accessible surface was colored by atom type. (C) The R.DpnI solvent accessible surface was colored according to the amino acid Eisenberg hydrophobicity scale (Eisenberg et al. JMB 1984, 179:125). Only the two central m6A:T base pairs are shown and part of the protein was omitted in lower left panels. The methyl groups are indicated with spheres of 2 Å radii.

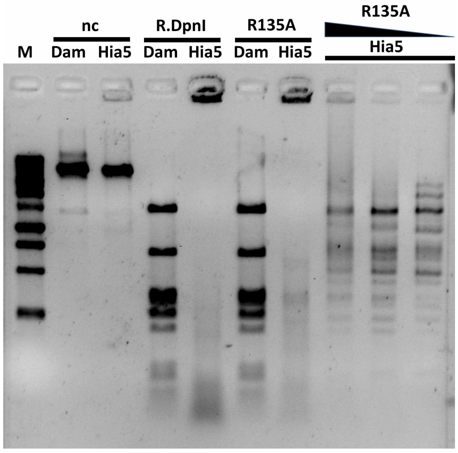

Figure S6. R.DpnI R135A variant sequence specificity assay. R.DpnI R135A variant mediated cleavage of Dam and Hia5-methylated pBR322 plasmid was compared with cleavage of the same substrates by WT R.DpnI. Enzyme concentration: 0.63 µM, pBR322 plasmid concentration: 13.77 nM. The wedge indicates decreasing concentration of R.DpnI R135A (0.13 µM, 0.063 µM, 0.0063 µM). M: molecular mass marker, nc: non-cleaved DNA, Dam: Dam-methylated plasmid, Hia5: Hia5-methylated plasmid isolated from a Dam+ *E. coli* strain.

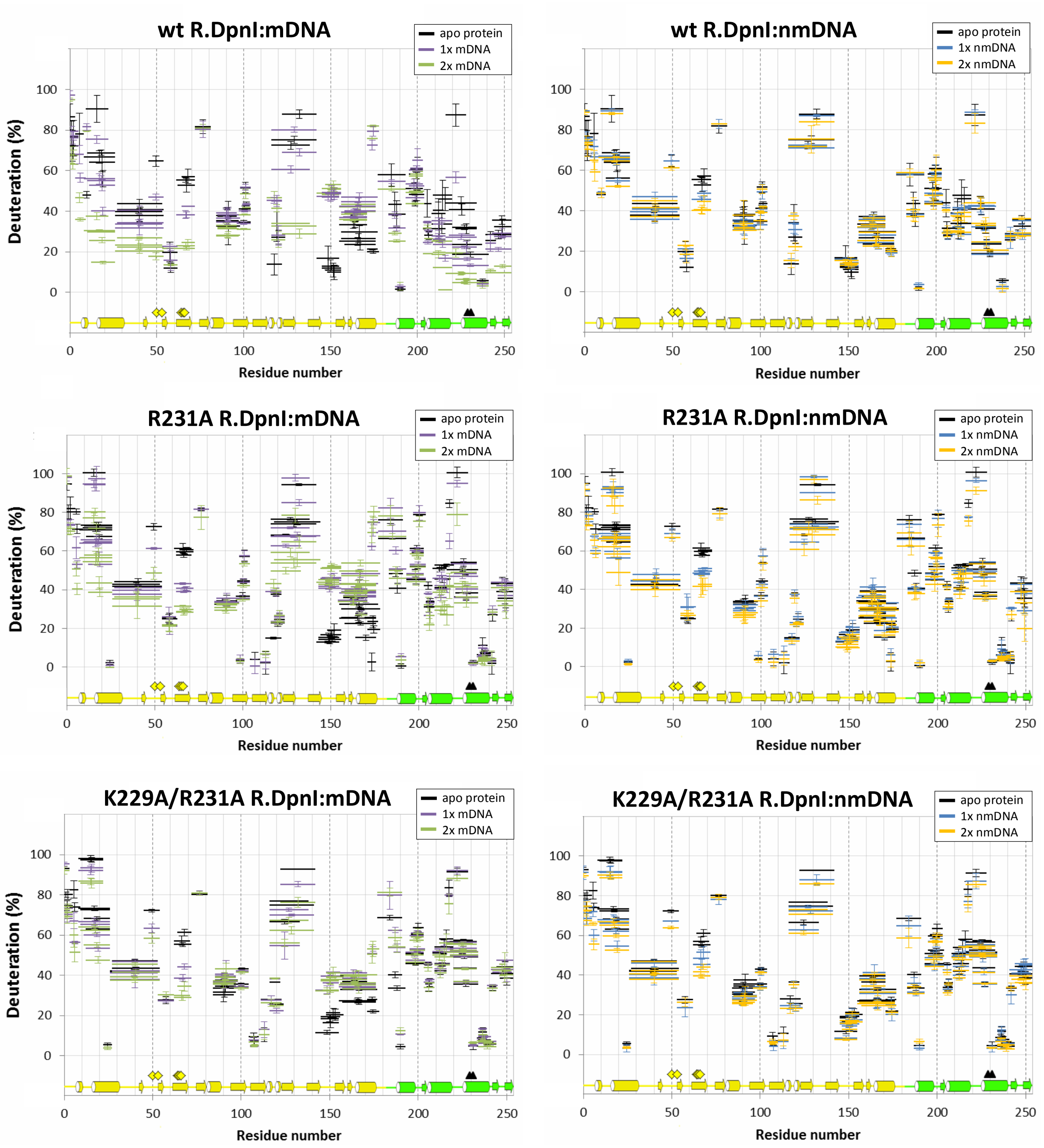

Figure S7: Deuteration of wild type R.DpnI and its R231A or K229A/R231A variants. The experiments have been carried out in the absence of DNA or in the presence of one or two equivalents of either methylated (left column) or nonmethylated (right column) DNA. Horizontal bars indicate peptides of corresponding length and sequence position. The vertical axis marks the fraction of deuterium after 10 s exchange time. Y-axis error bars come from two or three independent experiments. R.DpnI secondary structure is indicated at the bottom of each panel (catalytic domain in yellow, winged helix domain in green). Active site residues are marked by yellow diamonds and residues that have been mutated in winged helix domain to weaken DNA binding are indicated by black triangles.

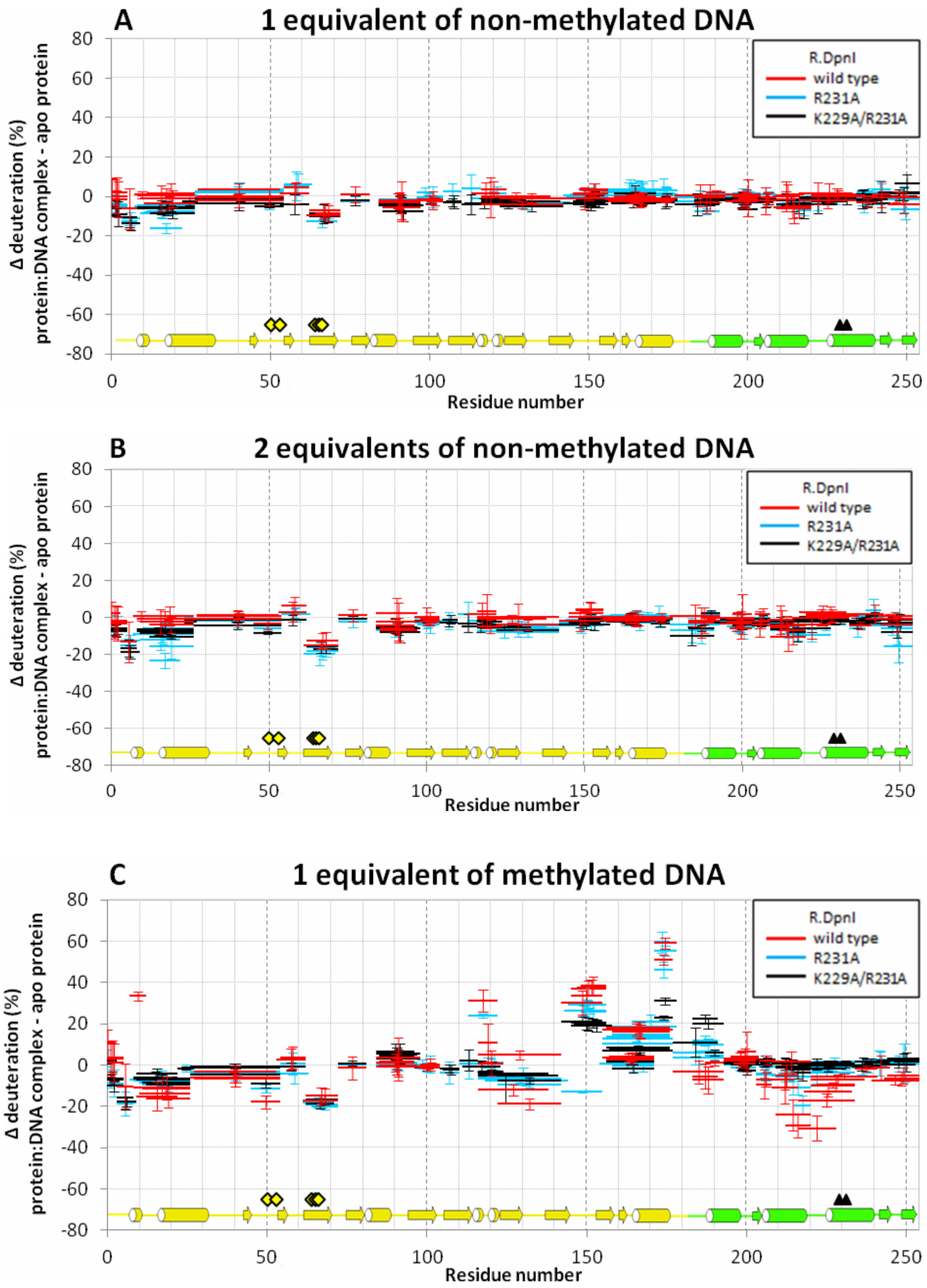

Figure S8: Deuteration of R.DpnI-DNA complex calculated as a difference versus the apo protein. (A) 1 equivalent of non-methylated DNA, (B) 2 equivalents of non-methylated DNA and (C) 1 equivalent of methylated DNA were added to wild type R.DpnI (red), R231A R.DpnI variant (blue) or K229A/R231A R.DpnI variant in (black).

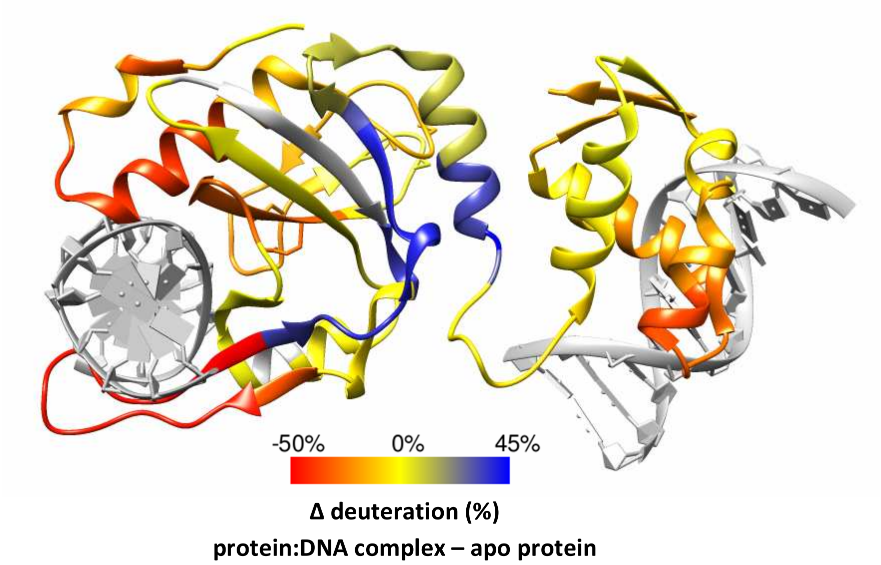

Figure S9: H**ydrogen/deuterium exchange rates mapped on the R.DpnI crystal structure**. A change of deuteration (10 s exchange time) upon addition of 2 molar equivalents of methylated DNA to wild type R.DpnI is calculated for each amino acid as an average of all peptides in which it is present. The structure is color coded as follows: red, strongly protected after addition of DNA; yellow, unchanged; blue, flexible after addition of DNA. Gray color represents regions not covered by peptic peptides in the sequence.

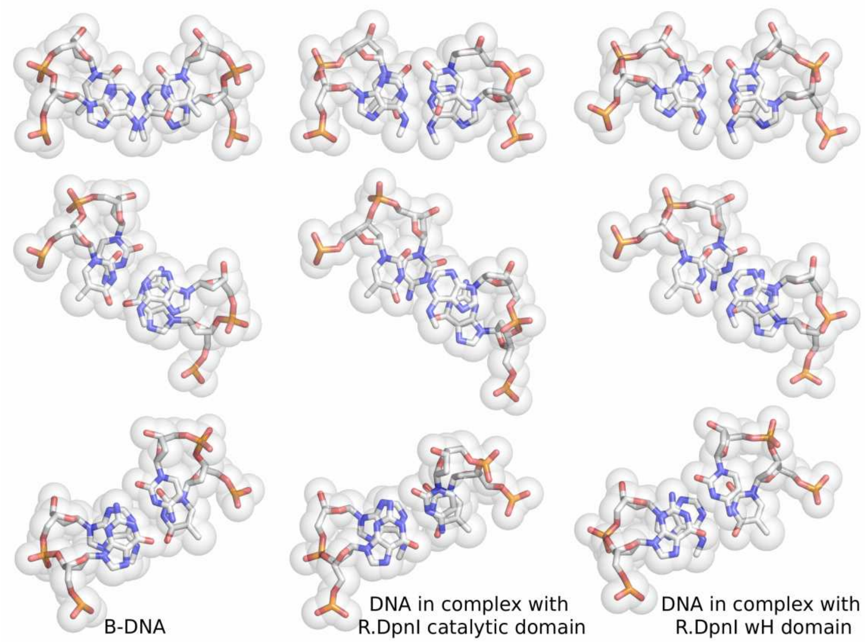

Figure S10: Stacking effects of 6-methyladenine with the neighboring DNA bases. The three columns depict the environment of the m6A:T pairs in B-DNA (left), in DNA in complex with R.DpnI catalytic (middle) and winged helix (right) domains. The panels illustrate the stacking of the two central base pairs of R.DpnI Gm6ATC target sequence with each other (top row) and of each m6A:T pair with the flanking G:C base pairs (middle and bottom rows).
